# Validity of CSF alpha-synuclein to predict psychosis in prodromal Alzheimer's disease

**DOI:** 10.3389/fneur.2023.1124145

**Published:** 2023-05-24

**Authors:** Sonia Monge-García, María-Salud García-Ayllón, José Sánchez-Payá, Ruth Gasparini-Berenguer, María-Ángeles Cortés-Gómez, Javier Sáez-Valero, José-Antonio Monge-Argilés

**Affiliations:** ^1^Instituto de Investigación Sanitaria y Biomédica de Alicante (ISABIAL), Alicante, Spain; ^2^Hospital General Universitario de Elche, FISABIO,Unidad de Investigación, Valencia, Spain; ^3^Instituto de Neurociencias de Alicante, Universidad Miguel Hernández-CSIC, Sant Joan d'Alacant, Spain; ^4^Unidad de Investigación, Hospital General Universitario de Elche, Fundación para el Fomento de la Investigación Sanitaria y Biomédica de la Comunitat Valenciana (FISABIO), Elche, Spain; ^5^Servicio de Medicina Preventiva, Hospital General Universitario Dr. Balmis, Alicante, Spain; ^6^Servicio de Neurología, Hospital General Universitario Dr. Balmis, Alicante, Spain

**Keywords:** psychosis, Alzheimer, alpha-synuclein, CSF, biomarkers

## Abstract

**Background:**

Alzheimer's disease (AD) accompanied by psychotic symptoms (PS) has a poor prognosis and may be associated with imbalances in key neural proteins such as alpha-synuclein (AS).

**Aim:**

The aim of the study was to evaluate the diagnostic validity of AS levels in the cerebrospinal fluid (CSF) as a predictor of the emergence of PS in patients with prodromal AD.

**Materials and methods:**

Patients with mild cognitive impairment were recruited between 2010 and 2018. Core AD biomarkers and AS levels were measured in CSF obtained during the prodromal phase of the illness. All patients who met the NIA-AA 2018 criteria for AD biomarkers received treatment with anticholinesterasic drugs. Follow-up evaluations were conducted to assess patients for the presence of psychosis using current criteria; the use of neuroleptic drugs was required for inclusion in the psychosis group. Several comparisons were made, taking into account the timing of the emergence of PS.

**Results:**

A total of 130 patients with prodromal AD were included in this study. Of these, 50 (38.4%) met the criteria for PS within an 8-year follow-up period. AS was found to be a valuable CSF biomarker to differentiate between the psychotic and non-psychotic groups in every comparison made, depending on the onset of PS. Using an AS level of 1,257 pg/mL as the cutoff, this predictor achieved at least 80% sensitivity.

**Conclusion:**

To our knowledge, this study represents the first time that a CSF biomarker has shown diagnostic validity for prediction of the emergence of PS in patients with prodromal AD.

## Introduction

The diagnosis of Alzheimer's disease (AD) must be restricted to people who exhibit positive biomarkers along with specific disease phenotypes (1). Behavioral and psychological symptoms are close to universally present in AD patients, and may include psychotic symptoms (PS) (2).

Psychotic symptoms are defined as the presence of delusions and/or hallucinations (2, 3), these being the most widely used diagnostic criteria for psychosis, as proposed by Jeste and Finkel (4) and revised by Cummings et al. (3). Over half of people with AD experience PS during their illness (5, 6). In particular, systematic reviews indicate a cross-sectional prevalence of approximately 40%, although it is recognized that lower rates occur in community populations and higher rates in clinical settings (2).

Psychotic symptoms have been found to be associated with rapid cognitive decline in AD (7, 8), and their emergence may represent a distinct phenotype (9). However, the association between AD psychosis and cognitive decline does not appear to be attributable to factors such as age at onset of AD, disease duration, sex, race, education, or family psychiatric history (10). PS are also associated with more rapid progression of functional impairment, hospital admission, earlier admission to institutional care, and increased mortality (11–15). Some studies have suggested that a sharper trajectory of decline occurs among people who develop PS, even before the onset of PS (8, 12, 16, 17). The use of antipsychotic drugs to treat PS in dementia is associated with greater mortality (18) and with adverse events (19).

This evidence suggests that a different underlying biological and/or genetic predisposition may be present in these individuals, and that the occurrence of PS represents a more severe AD phenotype (20); currently, however, there are no methods for predicting the occurrence of psychosis in AD patients.

The postulated neural mechanisms of AD psychosis include disturbances in cholinergic muscarinic receptors and altered concentrations of serotonin, tau protein, kalirin, and dopamine receptors (2). AD may be associated with selective alterations in dopamine receptor density (21, 22). Overexpression of wild-type alpha-synuclein (AS) decreases dopamine neurotransmission (23), and dopamine modifies aggregation of AS in the nervous system, resulting in greater abundance of AS oligomers (24). Lewy bodies, which are insoluble aggregates composed mainly of phosphorylated AS, are found in approximately 30%−50% of people with AD (25, 26); their presence contributes to the risk of psychosis and excess cognitive burden (21).

Various authors have published reports of alterations in CSF levels of AS in patients with AD, including in the prodromal phase of the illness (27–38). Increased CSF levels of AS have been found in patients with prodromal AD compared with controls and in patients with MCI attributable to Lewy body disease. Notably, however, a small subgroup of patients with MCI-AD and PS have been found to display low levels of CSF AS (37).

This study aimed to extend the sample size with a large cohort and to estimate the diagnostic validity of CSF levels of AS as a predictor of the emergence of PS in patients with prodromal AD.

## Materials and methods

### Study design and participants

This single-center retrospective cohort study included patients with mild cognitive impairment (MCI) in accordance with the Petersen criteria (39). These patients were recruited from the outpatient dementia consultation clinic at the Neurology Service of the Dr. Balmis General University Hospital (Alicante, Spain) between 2010 and 2018.

Patients were included in the study if they were aged over 55 years; had concordant clinical and neuropsychological diagnoses; exhibited a positive profile for AD biomarkers in their CSF (40); had an MMSE score ≥ 22, IQCODE score < 80, Barthel index ≥ 90, Neuropsychiatric Inventory (NPI) score < 8 (41), and Lawton–Brody scale (IADL) score ≥ 4; underwent clinical follow-up for > 2 years; and were treated with an anticholinesterasic drug following the diagnosis of prodromal AD (42). Informed consent was obtained from patients before their participation in this study and before lumbar puncture (LP) was performed. Patients who had dementia or other neurological, psychiatric, or medical diseases that could contribute to cognitive deterioration or psychosis according to the criteria established by Cummings et al. (3) at the time of inclusion; patients were also excluded if they had an UPDRS III score > 4 at inclusion, had an MRI Fazekas scale score >2 (43), were receiving anticoagulant therapy, did not provide informed consent, had a Yesavage score >5 for depression, or had a Pittsburgh sleep quality index > 7 (44).

All patients underwent physical and neurological examination, neuropsychological studies, cerebral magnetic resonance imaging, blood tests, and LP.

The NIA-AA criteria were used to evaluate conversion of MCI to AD (42). A control group was included, consisting of patients with acute or chronic headache (*n* =12) or pain syndrome (*n* =7) who did not undergo cognitive decline during the follow-up period.

*APOE* genotype was available for only 81 of the patients, because this was not technically possible to obtain during the first 5 years of recruitment.

### Procedures

Enrolled patients were evaluated every 6–12 months to check for the development of clinical dementia criteria (42). All the patients with AD met the dementia criteria within 2 years after LP and had been receiving anticholinesterasic treatment since the diagnosis of prodromal AD.

Psychotic symptoms were considered to be present when the patient scored >9 on the hallucinations + delusions (F x S) items of the NPI test (41), after exclusion of acute illness, delirium, or recent changes in treatment (3). No pharmacologic media had been used previously in any cases. With the agreement of the caregivers, neuroleptic treatment was initiated at a minimally effective dose.

### CSF collection

All CSF samples were obtained between 10:00 and 14:00. LP was performed by a neurologist using a 20 × 3.5-gauge needle. CSF was collected in standard polypropylene tubes, centrifuged for 10 min at 1,500 g, and then aliquoted in propylene tubes. Samples were stored at −80°C. Only samples with < 50 red blood cells/μL were included (28).

### Measurement of core CSF biomarkers of AD

Aβ_42_, total tau (T-tau), and phosphorylated tau 181 (p-tau_181_) were measured via commercial ELISA (Innotest, Innogenetic/Fujirebio, Ghent, Belgium) following the manufacturer's instructions. Assays were tested blind with respect to clinical diagnosis 6 months after LP.

Aβ_42_ > 800 pg/mL, T-tau < 350 pg/mL, and p-tau_181_ < 56,5 pg/mL were considered normal values. Patients were considered to have an AD CSF profile when at least Aβ_42_ and p-tau_181_ were abnormal, as per the 2018 NIA-AA criteria (40).

### Measurement of CSF AS

AS levels in the CSF were measured using the LEGEND MAX human AS ELISA kit with a precoated plate (BioLegend, San Diego, CA, USA) according to the manufacturer's instructions. Assays were performed in May 2021 in triplicate and blinded with respect to clinical diagnosis. This assay has previously been validated in a Europe-wide inter-laboratory study (45). Since a higher inter-assay CV was observed when the analysis was performed with different kit batches, all samples were analyzed with plates from the same batch, and one CSF sample was measured with each of the plates for standardization of AS levels between plates. CSF samples from each of the different groups were included on each plate. Luminescence detection was carried out using a BMG Labtech LUMIstar Optima.

### Statistical analysis

The Kolmogorov–Smirnov test was used to analyze the distribution of each quantitative variable. The Student's *t*-test (for parametric variables) and the Mann–Whitney *U* test (for non-parametric variables) were used to compare groups and subgroups. The chi-squared test or Fisher's exact test was used for qualitative variables.

Receiver operating characteristic (ROC) curve analysis was performed to determine the optimal cutoff for prediction of AS and the associated area under the curve (AUC). The optimal cutoff value was defined accounting for the highest sensitivity and specificity. Following this, the sensitivity, specificity, positive predictive value (PPV), and negative predictive value (NPV) for AS with the determined cutoff point were all calculated. In all hypothesis tests, a *p* ≤ 0.05 was determined to represent statistical significance. Correlations between motor/cognitive scores and AS levels and other CSF biomarkers in the diagnostic groups and subgroups were examined using Spearman's rho. The statistical package SPSS 21.0 was used for statistical analyses.

### Ethical approval

The study was approved by the Ethics Committees of the Dr. Balmis General University Hospital (ref. number: PI2020/250) and the Universidad Miguel Hernández (ref. number: PRL.IN.JSV.01.21).

## Results

### Population included

**Figure 1:**
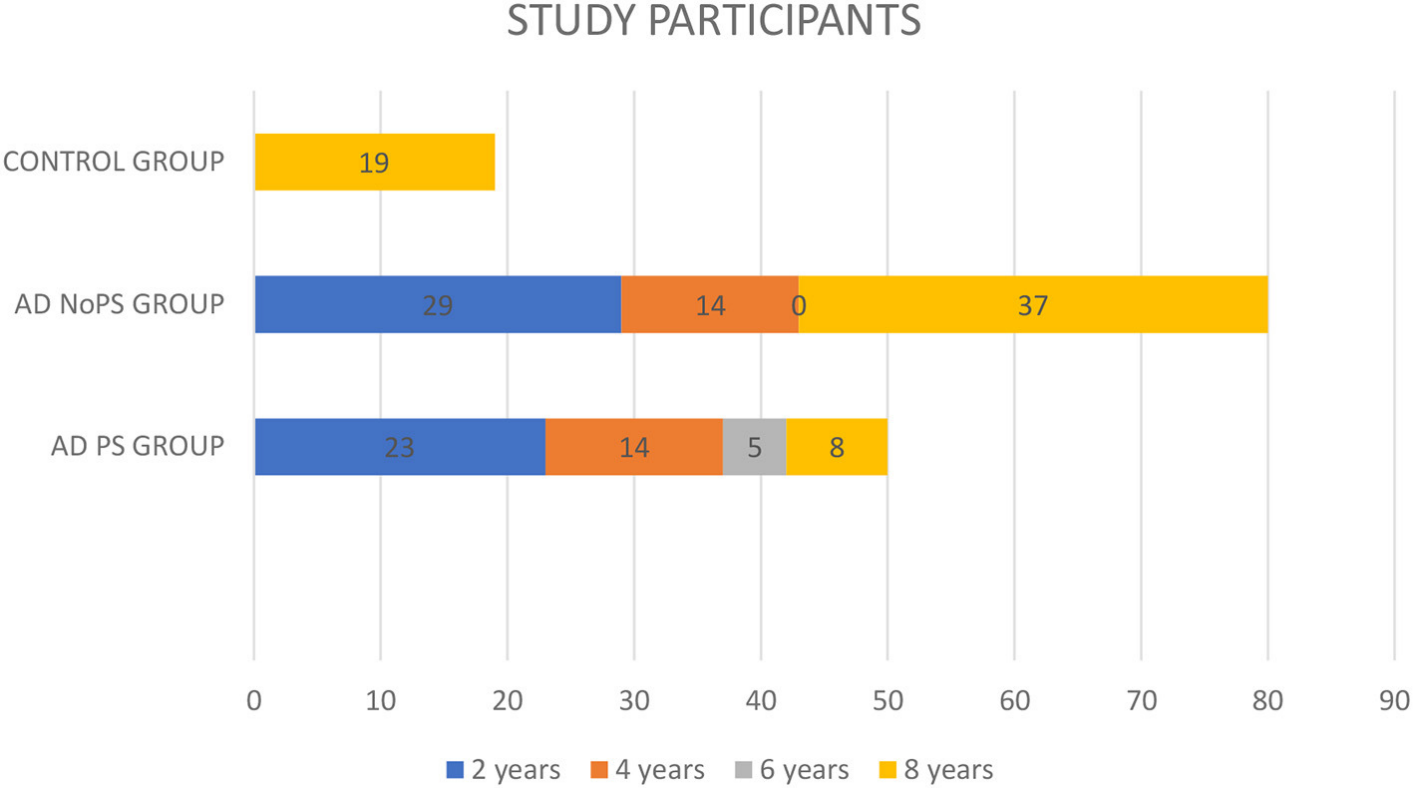
Follow-up of the study participants.

Figure 1 shows the distribution of patients across each group. A total of 50 patients with prodromal AD (38.4%) had developed PS (AD-PS group) 8 years after their initial diagnosis, with 37 of these (28%) having done so within the first 4 years after diagnosis.

The study also included 80 patients without PS (AD-No PS group): 37 (28%) remained free of PS over 8 years of follow-up. However, the follow-up period was < 4 years for 43 patients (33%) in this group. The minimum follow-up period was 2 years.

### Comparison of the psychosis group (AD-PS) with the non-psychosis group (AD-No PS)

The AD-PS group exhibited lower values for AS (*p* < 0.0001), the ratio of AS/p-tau_181_ (*p* < 0.006), p-tau_181_ levels (*p* < 0.01), and Aβ_42_ levels (*p* < 0.02) (Table 1, Supplementary Table 1). Clinically, the AD-PS group had lower NPI scores (*p* < 0.01) and UPDRS III scores (*p* < 0.007). No other differences were identified across the remaining variables. Correlation coefficients representing the associations between motor/cognitive scores and AS levels and other CSF biomarkers in the diagnostic groups are presented in Table 2. In both groups, CSF level of AS was positively correlated with T-tau (AD-PS group: ρ = 0.55, *P* < 0.0001; AD-No PS group: ρ = 0.41, *P* < 0.01), p-tau_181_ (AD-PS group: ρ = 0.56, *P* < 0.0001; AD-No PS group: ρ = 0.57, *P* < 0.001), NPI score (AD-PS group: ρ = 0.52, *P* < 0.01; AD-No PS group: ρ = 0.5, *p* < 0.05), and UPDRS (III) score (AD-PS group: ρ = 0.51, *P* < 0.01; AD-No PS group: ρ = 0.48, *P* < 0.05).

**Table 1:** Significant differences between groups and subgroups.

**Groups and subgroups compared**	**Clinical differences**	**Test differences**	**Differences in CSF biomarkers**
AD-PS (*n =* 50) vs. AD-NO PS (*n =*80)	NPI > AD-PS GROUP UPDRS III > AD-PS GROUP	————-	AS AS/p-TAU P-TAU_181_ Aβ_42_
AD- PS (*n =*50) vs. AD-NO PS at 8 years (*n =*37)	NPI > AD-PS GROUP UPDRS III > AD-PS GROUP	APOE genotype	AS P- TAU_181_
AD-PSY at 4 years (*n =*37) vs. AD- NO PS at 4 years (*n =*43)	MMSE > AD-NO PS GROUP UPDRS III > AD-PS GROUP IADL > AD-NO PS GROUP	FAZEKAS (+2 IN AD-PS GROUP) APOE (+ % ε4 carriers in AD-NO PS GROUP)	AS Aβ_42_ AS/p-TAU_181_ P- TAU_181_
AD- PS at 2 years (*n =* 23) vs. AD-NO PS at 2 years (*n =* 29)	NPI > AD-PS GROUP IADL > AD-NO PS GROUP AMNESTIC MCI > AD-NO PS GROUP	APOE (+ % ε4 carriers in AD- NO PS GROUP.)	AS Aβ_42_ AS/p-TAU_181_
AD- NO PS (*n =*80) vs. CONTROL (*n =*19)	PITTSBURGH SLEEP Q.I. > CONTROL GROUP IADL > CONTROL GROUP MMSE > CONTROL GROUP IQCODE > AD-NO PS GROUP DIABETES > CONTROL GROUP	APOE genotype (+ % ε4 carriers in AD- NO PSYCHOSIS GROUP)	All CSF biomarkers

**Table 2A:** Spearman correlations between motor/cognitive scores and AS levels and other CSF biomarkers in the diagnostic groups and subgroups. Psychosis group (AD-PS).

	**ρ value**	***P* value**
Aβ_42_ /AS	−0.42	0.7
T-tau/AS	0.55	0.0001
p-tau_181_/AS	0.56	0.0001
MMSE/AS	0.43	0.5
NPI/AS	0.52	0.01
UPDRS (III)/AS	0.51	0.01

**Table 2B:** No-psychosis group (AD-No PS).

	**ρ value**	***P* value**
Aβ_42_/AS	−0.32	0.6
T-tau/AS	0.41	0.01
p-tau_181_/AS	0.57	0.001
MMSE/AS	0.42	0.6
NPI/AS	0.5	0.05
UPDRS (III)/AS	0.48	0.05

**Table 2C:** AD no-psychosis group at 8-year follow-up.

	**ρ value**	***P* value**
Aβ_42_/AS	−0.24	0.5
T-tau/AS	0.45	0.01
p-tau_181_/AS	0.52	0.001
MMSE/AS	0.39	0.6
NPI/AS	0.48	0.05
UPDRS (III)/AS	0.52	0.05

**Table 2D:** AD no-psychosis group at 4-year follow-up.

	**ρ value**	***P* value**
Aβ_42_/AS	−0.31	0.7
T-tau/AS	0.51	0.01
p-tau_181_/AS	0.57	0.001
MMSE/AS	0.36	0.7
NPI/AS	0.45	0.05
UPDRS (III)/AS	0.49	0.05

**Table 2E:** AD no-psychosis group at 2-year follow-up.

	**ρ value**	***P* value**
Aβ_42_/AS	−0.4	0.5
T-tau/AS	0.43	0.01
p-tau_181_/AS	0.51	0.001
MMSE/AS	0.38	0.6
NPI/AS	0.46	0.05
UPDRS (III)/AS	0.48	0.05

**Table 2F:** AD psychosis group at 4-year follow-up.

	**ρ value**	***P* value**
Aβ_42_/AS	−0.32	0.6
T-tau/AS	0.4	0.01
p-tau_181_/AS	0.52	0.001
MMSE/AS	0.4	0.6
NPI/AS	0.42	0.05
UPDRS (III)/AS	0.52	0.01

**Table 2G:** AD psychosis group at 2-year follow-up.

	**ρ value**	***P* value**
Aβ_42_/AS	−0.42	0.7
T-tau/AS	0.38	0.01
p-tau_181_/AS	0.49	0.001
MMSE/AS	0.35	0.8
NPI/AS	0.4	0.05
UPDRS (III)/AS	0.48	0.05

AS, alpha-synuclein; Aβ_42_, Aβ_42_ protein; T-tau, total tau protein, p-tau_181_, p-tau_181_ protein; MMSE, Mini Mental State Examination; NPI, Neuropsychiatric Inventory; UPDRS (III), Unified Parkinson's Disease Rating Scale.

### Comparisons between subgroups

Comparisons between subgroups were carried out to assess the predictive value of the biomarkers and clinical parameters for PS during follow-up.

### Comparison of the AD-PS group with the AD-No PS subgroup at 8 years of follow-up

As compared with the AD-no PS subgroup 8 years after diagnosis (n = 37), the AD-PS group (n = 50) still exhibited lower AS levels (*p* < 0.006) and p-tau_181_ (*p* < 0.01), and higher scores on the NPI (*p* < 0.001) and the UPDRS III (*p* < 0.002). No other differences were found in the remaining variables, except in relation to *APOE* genotype (*p* < 0.001); however, these data were only available for a subset of patients (only 19 patients from the AD-PS group and two from the AD-No PS subgroup 8 years after diagnosis) (Table 1, Supplementary Table 2). Correlation coefficients representing the associations between motor/cognitive scores and AS levels and other CSF biomarkers in the diagnostic groups and subgroups are presented in Table 2. In the AD-No PS subgroup with 8 years of follow-up, CSF level of AS CSF was positively correlated with T-tau (ρ = 0.45, *p* < 0.01), p-tau_181_ (ρ = 0.52, *p* < 0.001), NPI score (ρ = 0.48, *p* < 0.05), and UPDRS (III) score (ρ = 0.52, *p* < 0.05) (Tables 2A–C).

### Comparison of the AD-PS subgroup with the AD-No PS subgroup at 4 years of follow-up

In a comparison of the subgroups limited to those patients with at least 4 years of follow-up, the AD-PS subgroup (*n* = 37) exhibited lower levels of AS (*p* < 0.001), Aβ_42_ (*p* < 0.004), and p-tau_181_ (*p* < 0.01) and a lower ratio of AS/p-tau_181_ (*p* < 0.02) as compared with the AD-No PS subgroup (n = 43). Clinically, the AD-PS subgroup had lower MMSE (*p* < 0.01) and IADL scores (*p* < 0.02) and higher UPDRS III scores (*p* < 0.04). Finally, the proportion of patients in this subgroup with the APOE- ε4 genotype was lower (*p* < 0.05; analysis limited to 61 of 80 patients), and patients in this subgroup had higher Fazekas MRI scores for white matter pathology (*p* < 0.02). No other differences were found in the remaining variables (Table 1, Supplementary Table 3). Correlation coefficients representing the associations between motor/cognitive scores and AS levels and other CSF biomarkers in the diagnostic subgroups are presented in Tables 2D, F. In both subgroups, CSF level of AS was positively correlated with T-tau (AD-PS subgroup: ρ = 0.4, *p* < 0.01; AD-No PS subgroup: ρ = 0.51, *p* < 0.01), p-tau_181_ (AD-PS subgroup: ρ = 0.52, *p* < 0.001; AD-No PS subgroup: ρ = 0.57, *p* < 0.001), NPI score (AD-PS subgroup: ρ = 0.42, *p* < 0.05; AD-No PS subgroup: ρ = 0.45, *p* < 0.05), and UPDRS (III) score (AD-PS subgroup: ρ = 0.52, *p* < 0.01; AD-No PS subgroup: ρ = 0.49, *p* < 0.05).

### Comparison of the AD-PS subgroup with the AD-No PS subgroup at 2-year follow-up

Since we were investigating the prognostic value of the biomarkers, we also compared subgroups at the shortest period of follow-up: that is, patients with only a 2-year follow-up period. This AD-PS subgroup (*n* =23) exhibited lower levels of AS (*p* < 0.002) and Aβ_42_ (*p* < 0.02) and a lower ratio of AS/p-tau_181_ (*p* < 0.05) as compared with the AD-No PS subgroup (*n* =29). The AD-PS subgroup also had a lower rate of amnestic MCI (*p* < 0.02), a lower proportion of patients with the *APOE*- ε4 genotype (*p* < 0.05, data limited to 42 of 52 patients), and lower IADL scores (*p* < 0.02), but they had higher NPI scores (*p* < 0.02). No other differences were found in the remaining variables (Supplementary Table 4). Correlation coefficients representing the associations between motor/cognitive scores and AS levels and other CSF biomarkers in the diagnostic subgroups are presented in Table 2. In both subgroups, CSF level of AS was positively correlated with T-tau (AD-PS subgroup: ρ = 0.38, *p* < 0.01; AD-No PS subgroup: ρ = 0.43, *p* < 0.01), p-tau_181_ (AD-PS subgroup: ρ = 0.49, *p* < 0.001; AD-No PS subgroup: ρ = 0.51, *p* < 0.001), NPI score (AD-PS subgroup: ρ = 0.40, *p* < 0.05; AD-No PS subgroup: ρ = 0.46, *p* < 0.05), and UPDRS (III) score (AD-PS subgroup: ρ = 0.48, *p* < 0.01; AD-No PS subgroup: ρ = 0.48, *p* < 0.05) (Tables 2E, G).

### Comparison of the AD-No PS group with the control group

In comparison to the control group, the AD-No PS group exhibited higher levels of AS (*p* < 0.04), T-tau (*p* < 0.0001), and p-tau_181_ (*p* < 0.0001); higher ratios of T-tau/Aβ_42_ (*p* < 0.0001) and p-tau_181_/Aβ_42_ (*p* < 0.0001); lower levels of Aβ_42_ (*p* < 0.0001); and a lower AS/p-tau_181_ ratio (*p* < 0.001). Clinically, they had higher IQCODE scores (*p* < 0.0001) and a higher incidence of the *APOE*- ε4 genotype (*p* < 0.01; data limited to 61 of 99 patients), but had lower scores on the Pittsburgh sleep quality index (*p* < 0.02), MMSE (*p* < 0.0001), and IADL (*p* < 0.001). No other differences were found (Table 1, Supplementary Table 5).

### ROC curves

ROC curves were defined in order to assess the diagnostic value of AS. Taking an AS level of 1,257 as the cutoff, the use of this threshold showed sensitivity equal to or >80% in differentiating between the groups and subgroups analyzed (Table 3). The NPV reached 80% in differentiating between the AD-PS and AD-No PS groups (Table 3).

**Table 3:** ROC curves for AS level at a cutoff of 1,257 pg/ml as a differentiator between AD groups and subgroups.

**Groups and subgroups compared**	**AUC [95% CI]**	**Sensitivity (%)**	**Specificity (%)**	**PPV (%) [95% CI]**	**NPV (%) [95% CI]**
AD-PS (*n =* 50) vs. AD- NO PS (*n =* 80)	0.71 [0.61–0.80]	80	46	48 [39.3–57]	80 [74–87]
AD-PS (*n =* 50) vs. AD-NO PS 8 years (*n =* 37)	0.67 [0.55–078]	80	35	63 [56.2–71]	57 [50.2–66]
AD-PSY at 4 years (*n =* 37) vs. AD- NO PS at 4 years (*n =* 43)	0.73 [0.61–0.83]	81	47	60 [53–69.3]	73 [67.2–79]
AD-PS at 2 years (*n =* 23) vs. AD-NO PS at 2 years (*n =* 29)	0.75 [0.62–0.88]	83	45	61 [54–69.5]	75 [69–84.2]

AUC, area under the curve; PPV, positive predictive value; NPV, negative predictive value; CI, confidence interval.

## Discussion

This study indicated that AS is a valuable CSF biomarker for prediction of PS in a prodromal AD patient cohort. Among the other CSF biomarkers, p-tau_181_, AS/p-tau_181_ ratio, and Aβ_42_ also reached the threshold, but displayed lower diagnostic validity for this purpose.

The presence of psychotic symptoms in AD is reportedly associated with AS and/or tau cerebral pathologies (8). The pathogenic relationship between AS and PS is recognized because of their common implication of cerebral dopamine levels (23, 24). Increased AS affects dopamine neurotransmission at multiple levels, particularly in decreasing dopamine synthesis (23). Moreover, dopamine influences the aggregation of AS in the nervous system, which results in AS oligomers and unique dopamine-induced oligomeric conformations (24). Selective alterations in dopamine receptor density have been found *postmortem* and *in vivo* in patients with AD and PS; these alterations may be associated with distinct clinical profiles (21, 22).

In the present study, lower CSF levels of AS were found in the AD-PS group than in the AD-No PS group. Such a decrease may be associated with the cerebral deposition of AS, forming Lewy bodies in alpha-synucleinopathies (46, 47). A number of studies have reported AS pathology in approximately 50% of autopsied patients with AD (25, 36). Patients with AD and AS tend to exhibit amplified deterioration, typically enduring more severe symptoms and shorter duration of survival (47), which may contribute to a distinctive clinical profile within AD (9, 22). Evidence from other studies suggests that AS might be involved in the development of AD from the very early stages of Aβ pathology formation (48), as well as tau hyperphosphorylation (26). These data suggest that AS is involved in the pathophysiology of AD (26). Early identification of this condition in patients with AD, including quantification of CSF AS, should enable provision of a better treatment plan and improvements in prognosis (37).

The AD-No PS group exhibited higher levels of AS than the control group, particularly during the first 4 years after diagnosis. These results were in line with those of previous studies, with short follow-up periods and without follow-up, than have taken PS into account in the clinical description or evolution (27–29, 32–36). The increase in AS observed in AD patients is based on evidence on elevated AS in the brain tissue of patients with AD (49) and/or the neuronal damage related to AD (33). This increase in CSF levels of AS in AD patients is associated with the accumulation of amyloid plaques (26) and tau proteins (36, 50, 51). These results are in accordance with the lower levels of Aβ_42_, higher levels of p-tau_181_, and higher AS/p-tau_181_ ratio observed in the AD-No PS group in the present study. In a previous study, PS was found to be associated with tau phosphorylation abnormalities (8). Nevertheless, although that association should be female-specific, the incidence of PS in AD should be more likely among men because of the nature of AS pathology (8). To our knowledge, no gender differences have been described in the relationship of PS with Aβ_42_ protein and the ratio of AS/p-tau_181_.

In this study, the decrease in the aforementioned ratio among the AD-PS group was more attributable to lower levels of AS than to a clear decrease in the levels of p-tau_181_. Another independent report, on a longitudinal study of AD, has found that lower values for this ratio predict faster cognitive decline (35).

The sensitivity of AS in differentiating between the AD-PS and AD-No PS groups is notable, considering the ability to predict the occurrence of PS, which requires specific pharmacologic intervention. The emergence of PS is associated with endogenous and exogenous factors, including underlying biological and/or genetic predispositions in the individual (2). Regarding endogenous factors, of the dopaminergic factors mentioned above, certain alterations to the cholinergic (2, 52–56) and serotoninergic systems (57, 58) are related to the occurrence of PS in AD. In terms of exogenous factors, sleep quality, familial relationships with caregivers, medical antecedents, schooling, and capacity to engage in activities of daily living should influence the emergence of PS in AD (2). No significant differences at the time of inclusion were found between the AD groups on any of these variables, including demographic and radiological data, indicating the high level of homogeneity between the groups included in this study.

Clinical NPI and UPDRS (III) scores increased among the PS group over the period following inclusion. These data may be concordant with abnormal dopaminergic status, which is probably related to the amount of cortical and subcortical Lewy bodies (59). To date, the concept of mixed AD + dementia with Lewy bodies is accepted in neuropathological settings as a difficult clinical diagnosis, with a lack of biomarkers to assist identification (60). Quantification of CSF level of AS may facilitate this objective (61). Positive correlations were observed between AS levels, such scores, and tau protein levels in both AD groups and in the subgroups. These findings in the initial stages of AD support the involvement of AS in the pathogenesis of AD (27, 32, 52).

Alzheimer's disease is considered to be a clinical–biological entity (1). Our data and data presented in previous publications (27, 28, 32–37) support the added value of measurement of CSF levels of AS in further characterization of the CSF AD biomarker profile. The contradictory results published in some previous reports regarding the potential use of CSF level of AS as a diagnostic biomarker for AD may be attributable to various factors, including reduced sample size, uncertain diagnosis and/or uncontrolled follow-up, enrolment of patients at different stages of the disease, differences in age, and lack of control for blood contamination, among others (29, 62–65). The current AT (N) classification for a biological definition of AD has been defined as flexible, meaning that new biomarkers can be added when they become available (40), and there is a need for more biomarkers, such as AS, indicating other aspects of the mechanisms of the disease (66–69).

Beta-synuclein is another member of the synuclein family and is emerging as a reliable synaptic marker in CSF and blood for AD and prion disease (70, 71). In the study by Barba et al., increased levels of CSF AS were observed in pre-AD patients, but not in MCI-AD or dementia-AD. Beta-synuclein was elevated in all AD continuum subgroups. Elevated CSF levels of both beta-synuclein and AS in pre-AD may reflect the earliest synaptic dysfunction occurring in AD. Decreased AS levels may indicate the presence of α-synucleinopathy, whereas beta-synuclein concentrations are not influenced by the presence of synucleinopathy or by blood contamination, which instead affects AS measurements.

In this study, the handling of the samples was performed as per recommended operating procedures (72). CSF samples with < 50 red blood cells/μL were included (28), and the reagents used had been validated in a Europe-wide inter-laboratory study (45). These points of analytical and methodological validity are critical for the value of these results. Nevertheless, in relation to protein-misfolding cyclic amplification (PMCA) and real-time quaking-induced conversion (RT-QuIC), ultrasensitive protein amplification assays for the detection of misfolded protein aggregates could also offer diagnostic reliability for AS levels in CSF. In particular, RT-QuIC assay has demonstrated high specificity and sensitivity for detection of CSF AS aggregation in patients with synucleinopathies when compared to AD patients and controls (73–75). The possibility of analyzing the cholinergic, glutamatergic, and serotoninergic pathways with these techniques should be of great interest for completion of the study of PS in AD patients.

The main limitation of this study was the lack of neuropathological confirmation. Nevertheless, to our knowledge, the long clinical follow-up period of the patients was among the longest across all published studies. The clinical and biological data were very conclusive. However, the ability of caregivers to control behavioral disturbances was not tested, and a portion of the AD-No PS group had an incomplete period of follow-up in which the emergence of PS could be excluded. Finally, the lack of data on APOE genotype in patients recruited during the first 5 years of the study was another limitation.

In conclusion, the quantification of CSF AS in patients with prodromal AD allows for prediction of the emergence of PS during the subsequent 8 years with high sensitivity. In this regard, early identification will enable the provision of better treatment plans and improvements in prognosis. Future biomarker panels, including the biological study of cholinergic and serotoninergic pathways, will probably enable more complete prediction of PS in AD.

## Data availability statement

The original contributions presented in the study are included in the article/Supplementary material, further inquiries can be directed to the corresponding authors.

## Ethics statement

The studies involving human participants were reviewed and approved by Ethical Committee of University General Hospital Dr. Balmis. Alicante, Ref number: PI2020/250. The patients/participants provided their written informed consent to participate in this study.

## Author contributions

All authors listed have made a substantial, direct, and intellectual contribution to the work and approved it for publication.
