# Supplementary material for: Validity of CSF alpha-synuclein to predict psychosis in prodromal Alzheimer's disease
Source: Front Neurol. 2023 May 24;14:1124145. doi: 10.3389/fneur.2023.1124145 (PMC10244520; doi:10.3389/fneur.2023.1124145)
Supplement: Supplementary file 1 [file Data_Sheet_1.docx]

**TITLE: VALIDITY OF CSF ALPHA-SYNUCLEIN TO PREDICT PSYCHOSIS**

**IN PRODROMAL ALZHEIMER’S DISEASE.**

**JOURNAL NAME: INTERNATIONAL JOURNAL OF MOLECULAR SCIENCES**

**AUTHORS: Monge-García** Sonia (1), **García-Ayllón** María-Salud (2,3,4), **Sánchez-Payá** José (1,5), **Gasparini-Berenguer** Ruth (1), **Cortés-Gómez** María-Ángeles (2,3,4), **Sáez-Valero** Javier (1,3,4), **Monge-Argilés** José-Antonio (1,6)**.**

**CORRESPONDENCE:** JOSÉ-ANTONIO MONGE-ARGILÉS

e-mail [monge_jos@gva.es](mailto:monge_jos@gva.es)

or

MARÍA-SALUD GARCÍA-AYLLÓN

e- mail: ms.garcia@umh.es

**Table 1. Comparison AD-PSYCHOSIS GROUP versus AD-No PSYCHOSIS GROUP.**

|  | **AD-PS GROUP**  **(n= 50)** | **AD-NO PS GROUP**  **(n=80)** | ***p* value** |
| --- | --- | --- | --- |
| AGE mean ± SD | 72.9 ± 6.4 | 72.9 ± 7.1 | 0.95 |
| SEX M/F (% M) | 19/31(38) | 30/50 (37.5) | 1.0 |
| ANTECEDENTS  Diabetes (%)  Hypertension (%)  Hyperlipidemia (%)  Depression (%) | 9 (18)  21 (42)  30 (60)  10 (20) | 15 (18)  47 (58)  50 (63)  20 (25) | 0.9  0.07  0.8  0.5 |
| SCHOLLING YEARS  median [p25-p75] | 3 [1-7] | 3 [2-7] | 0.3 |
| START OF THE SYMPTOMS mean ± SD (months) | 19.1 ± 3.4 | 23.4 ± 4.3 | 0.07 |
| MMSE median [p25-p75] | 24 ]23-26] | 25 [24-26] | 0.1 |
| IQCODE median [p25-p75] | 70 [60-75] | 70 [60-70] | 0.9 |
| NPI median [p25-p75] | 4 [2-6] | 2 [2-6] | 0.01 |
| BARTHEL INDEX | 100 | 100 | 0.9 |
| IADL median [p25-p75] | 5 [4-7] | 6 [5-7] | 0.1 |
| FA DEMENTIA (%) | 8 (16) | 21 (26) | 0.1 |
| MEDIAL TEMPORAL ATROPHY  median [p25-p75] | 2 [0-2] | 2 [0-2] | 0.8 |
| FAZEKAS SCHELLE 0  1  2 | 21 (42)  18 (36)  11 (22) | 30 (38)  36 (45)  14 (17) | 0.5 |
| PSQI median [p25-p75] | 4 [2-6] | 4 [2-6] | 0.6 |
| LIVING  In couple (%)  Others (%) | 29 (58)  21 (42) | 49 (61)  31 (39) | 0.7 |
| AMNESTIC MCI (%) | 46 (92) | 79 (98) | 0.06 |
| UPDRS (III) median [p25-p75] | 0 [0-4] | 0 | 0.007 |
| APOE GENOTYPE  ε 3 (%)  ε 4 (%) | 10 (20)  9 (18) | 20 (25)  25 (31) | 0.5 |
| Aβ_42_ (pg/mL)  median (p25-p-75) | 532  (472-652) | 627  (508-701) | 0.02 |
| T-TAU (pg/mL)  median (p25-p-75) | 534  (337-718) | 534  (430-766) | 0.2 |
| P-TAU_181_ (pg/mL)  median (p25-p-75) | 75  (60-98) | 91  (71-118) | 0.01 |
| RATIO TTAU/Aβ_42_  median (p25-p-75) | 0.83  (0.71-1.35) | 0.88  (0.68-1.34) | 0.9 |
| RATIO P-TAU_181_/Aβ_42_  median (p25-p-75) | 0.14  (0.1-0.18) | 0.14  (0.11-0.2) | 0.4 |
| ALPHA-SYNUCLEIN (pg/mL)  median (p25-p-75) | 926  (754-1098) | 3577  (1646-5508) | 0.0001 |
| RATIO AS/P-TAU_181_  median (p25-p-75) | 10.5  (6.0-15.6) | 12.9  (8.6-21.0) | 0.006 |

**Table 2. Comparison AD-PSCHOSIS GROUP versus AD-NO PSYCHOSIS SUBGROUP INTO 8 YEARS FOLLOW-UP.**

|  | **AD-PS GROUP**  **(n= 50)** | **AD-NO PS GROUP**  **INTO 8 years (n=37)** | ***p* value** |
| --- | --- | --- | --- |
| AGE mean ± SD | 72.9 ± 6.4 | 74.8 ± 6.1 | 0.15 |
| SEX M/F (M %) | 19/31 (38) | 11/26 (29.7) | 0.5 |
| ANTECEDENTS  Diabetes (%)  Hypertension (%)  Hyperlipidemia (%)  Depression (%) | 9 (18)  21 (42)  30 (60)  10 (20) | 5 (13)  20 (54)  20 (54)  8 (21) | 0.5  0.2  0.5  0.8 |
| SCHOOLING YEARS  median [p25-p75] | 3 [1-7] | 2 [2-5] | 0.5 |
| START OF THE SYMPTOMS mean ± SD (months) | 19.1 ± 3.4 | 22.2 ± 4.1 | 0.1 |
| MMSE median [p25-p75] | 24 [23-26] | 25 [23-26] | 0.5 |
| IQCODE median [p25-p75] | 70 [60-75] | 65 [60-70] | 0.3 |
| NPI median [p25-p75] | 4 [2-6] | 2 [0-4] | 0.001 |
| BARTHEL INDEX | 100 | 100 | 0.5 |
| IADL median [p25-p75] | 5 [4-7] | 5 [5-7] | 0.5 |
| FA DEMENTIA (%) | 8 (16) | 5 (13) | 0.7 |
| MTA median [p25-p75] | 2 [0-2] | 1 [1-2] | 0.8 |
| FAZEKAS SCHELLE 0 (%)  1 (%)  2 (%) | 21 (42)  18 (36)  11 (22) | 17 (46)  10 (27)  10 (27) | 0.6 |
| PITTSBURG SLEEP Q. I.  median [p25-p75] | 4 [2-6] | 4 [2-6] | 0.7 |
| LIVING  In couple (%)  Others (%) | 29 (58)  21 (42) | 17 (46)  20 (54) | 0.2 |
| AMNESTIC MCI (%) | 46 (92) | 37 (100) | 0.07 |
| UPDRS III median [p25-p75] | 0 [0-4] | 0 [0-0] | 0.002 |
| APOE GENOTYPE  ε 3 (%)  ε 4 (%) | 10 (20)  9 (18) | 1 (2)  1 (2) | 0.001 |
| Aβ_42_ pg/mL  median (p25-p-75) | 532  (472-652) | 575  (489-676) | 0.4 |
| T-TAU pg/mL  median (p25-p-75) | 534  (337-718) | 550  (419-768) | 0.3 |
| P-TAU_181_ pg/mL  median (p25-p-75) | 75  (60-98) | 93  (75-118) | 0.01 |
| RATIO TTAU/Aβ_42_  median (p25-p75) | 0.83  (0.71-1.35) | 1.07  (0.68-1.45) | 0.5 |
| RATIO P-TAU_181_/Aβ_42_  median (p25-p75) | 0.14  (0.11-0.18) | 0.16  (0.12-0.23) | 0.1 |
| ALPHA-SYNUCLEIN pg/mL median (p25-p-75) | 842  (754-1098) | 1045  (408-7465) | 0.006 |
| RATIO AS/P-TAU_181_  median (p25-p75) | 10.56  (6.0-15.6) | 10.4  (7.2-21.6) | 0.2 |

**Table 3. Comparison AD-PSYCHOSIS SUBGROUP INTO 4 YEARS FOLLOW-UP versus AD-NO PSYCHOSIS SUBGROUP INTO 4 YEARS FOLLOW-UP.**

|  | **AD-PS GROUP**  **into 4 years (n=37)** | **AD-NO PS GROUP**  **into 4 years (n=43)** | ***P* value** |
| --- | --- | --- | --- |
| AGE mean ± SD | 72.4 ± 6.9 | 71.3 ± 7.5 | 0.3 |
| SEX (M/F) (% M) | 16/21 (43) | 19/24 (44) | 0.9 |
| ANTECEDENTS  Diabetes (%)  Hypertension (%)  Hyperlipidemia (%)  Depression (%) | 7 (19)  18 (48)  24 (65)  7 (19) | 9 (21)  26 (61)  29 (69)  12 (28) | 0.7  0.2  0.7  0.3 |
| SCHOOLING YEARS  median [p25-p75] | 3 [2-8] | 6.5 [2-7.2] | 0.2 |
| START OF THE SYMPTOMS mean ± SD | 20.1 ± 7.3 | 23.4 ± 5.2 | 0.06 |
| MMSE median [p25-p75] | 24 [23-26] | 25[24-27] | 0.01 |
| IQCODE median [p25-p75] | 70 [60-75] | 70 [65-75] | 0.9 |
| NPI median [p25-p75] | 4 [4-6] | 4 [2-6] | 0.1 |
| BARTHEL INDEX | 100 | 100 | 0.8 |
| IADL median [p25-p75] | 5 [4-7] | 6 [5-8] | 0.02 |
| FA DEMENTIA (%) | 7 (19) | 16 (38) | 0.06 |
| MTA median [p25-p75] | 2 [0-2] | 2 [0-2] | 0.7 |
| FAZEKAS SCHELLE 0 (%)  1 (%)  2 (%) | 13 (35)  14 (37)  10 (27) | 13 (31)  26 (61)  4 (7) | 0.02 |
| PITTSBURG SLEEP Q.I.  median [p25-p75] | 2 [2-5] | 3 [2-6.5] | 0.5 |
| LIVING  In couple (%)  Others (%) | 20 (54)  17 (46) | 31 (73)  12 (27) | 0.06 |
| AMNESTIC MCI (%) | 33 (89) | 41 (95) | 0.1 |
| UPDRS III median [p25-p75] | 0 (0-5) | 0 (0-0) | 0.04 |
| APOE GENOTYPE  ε 3 (%)  ε 4 (%) | 9 (27)  9 (27) | 19 (44)  24 (55) | 0.05 |
| Aβ_42_ pg/mL.  median (p25-p75) | 528  (456-637) | 646  (553-704) | 0.004 |
| T-TAU pg/mL.  median (p25-p75) | 512  (323-698) | 531  (442-764) | 0.1 |
| P-TAU_181_ pg/mL.  median (p25-p75) | 68  (59-89) | 88  (70-116) | 0.01 |
| RATIO T-TAU/ Aβ_42_  median (p25-p75) | 0.84  (0.7-1.3) | 0.87  (0.6-1.1) | 0.8 |
| RATIO P-TAU_181_/ Aβ_42_  median (p25-p75) | 0.14  (0.11-0.16) | 0.14  (0.10-0.18) | 0.6 |
| ALPHA-SYNUCLEIN pg/mL.  median (p25-p75) | 848  (459-1256) | 1394  (946-1965) | 0.001 |
| RATIO AS/P-TAU_181_  median (p25-p75) | 11.5  (7.5-16.6) | 15.0  (10.8-21.3) | 0.02 |

**Table 4. Comparison AD-PSYCHOSIS SUBGROUP INTO 2 YEARS FOLLOW-UP versus AD-NO PSYCHOSIS INTO 2 YEARS FOLLOW-UP.**

|  | **AD-PS GROUP**  **into 2 years (n=23)** | **AD-NO PS GROUP**  **into 2 years (n=29)** | ***P* value** |
| --- | --- | --- | --- |
| AGE mean ± SD | 71.6 ± 6.6 | 72.8 ± 7.4 | 0.4 |
| SEX M/F (% M) | 11/12 (47) | 13/16 (44) | 0.8 |
| ANTECEDENTS  Diabetes (%)  Hypertension (%)  Hyperlipidemia (%)  Depression (%) | 3 (13)  10 (43)  13 (56)  5 (21) | 6 (21)  16 (57)  20 (71)  7 (25) | 0.4  0.3  0.2  0.7 |
| SCHOOLING YEARS  median [p25-p75] | 3 [2-8] | 7 [2-7] | 0.6 |
| START OF THE SYMPTOMS  mean ± SD | 18.4 ± 4.3 | 22.7 ± 3.6 | 0.07 |
| MMSE median [p25-p75] | 24 [23-26] | 25 [23-27] | 0.1 |
| IQCODE median [p25-p75] | 70 [60-75] | 70 [65-73] | 0.4 |
| NPI median [p25-p75] | 6 [4-6] | 4 [2-6] | 0.02 |
| BARTHEL INDEX | 100 | 100 | 0.8 |
| IADL  median [p25-p75] | 5 [4-6] | 6 [5-7] | 0.02 |
| FA DEMENTIA (%) | 6 (26) | 10 (35) | 0.4 |
| MTA median [p25-p75] | 2 [0-2] | 2 [0-2] | 0.8 |
| FAZEKAS SCHELLE 0 (%)  1 (%)  2 (%) | 8 (35)  11 (47)  4 (17) | 10 (35)  15 (53)  4 (11) | 0.7 |
| PITTSBURG SLEEP Q.I.  median [p25-p75] | 2 [ 2-4] | 2 [2-6] | 0.9 |
| LIVING  In couple (%)  Others (%) | 13 (56)  10 (44) | 20 (71)  9 (29) | 0.2 |
| AMNESTIC MCI (%) | 19 (82) | 28 (100) | 0.02 |
| UPDRS III median [p25-p75] | 0 (0-5) | 0 | 0.9 |
| APOE GENOTYPE  ε 3 (%)  ε 4 (%) | 8 (34)  5 (21) | 15 (51)  14 (48) | 0.05 |
| Aβ_42_ pg/mL.  median (p25-p75) | 528  (468-684) | 658  (568-743) | 0.02 |
| T-TAU pg/mL.  median (p25-p75) | 574  (337-718) | 575  (463-989) | 0.1 |
| P-TAU_181_ pg/mL.  median (p25-p75) | 76  (61-94) | 89  (67-129) | 0.07 |
| RATIO TTAU/Aβ_42_ median (p25-p75) | 0.86  (0.73-1.3) | 0.93  (0.67-1.26) | 0.8 |
| RATIO P-TAU_181_/ Aβ_42_ median (p25-p75) | 0.14  (0.12-0.16) | 0.14  (0.1-0.19) | 0.9 |
| ALPHA-SYNUCLEIN pg/mL. median (p25-p75] | 992  (730-1254) | 3297  (847-5748) | 0.002 |
| RATIO AS/P-TAU_181_  median (p25-p75) | 11.5  (7.9-15.8) | 15.0  (10.8-21.7) | 0.05 |

**Table 5. Comparison CONTROL GROUP versus AD-NO PSYCHOSIS GROUP.**

|  | **CONTROL GROUP**  **(n=19)** | **AD-NO PS GROUP**  **(n=80)** | ***P* value** |
| --- | --- | --- | --- |
| AGE mean ± SD | 68.0 ± 7.1 | 72.9 ± 7.1 | 0.5 |
| SEX M/F (% M) | 7/12 (36.8) | 30/50 (37.5) | 0.9 |
| ANTECEDENTS  Diabetes (%)  Hypertension (%)  Hyperlipidemia (%)  Depression (%) | 8 (42)  13 (68)  13 (68)  9 (47) | 14 (18)  46 (58)  49 (62)  20 (25) | 0.02  0.4  0.6  0.06 |
| SCHOOLING YEARS median [p25-p75] | 7 [2-10] | 3 [2-7] | 0.2 |
| START OF THE SYMPTOMS  mean ± SD | ---------------- | 23.4 ± 4.3 | -------------- |
| MMSE median [p25-p75] | 28 [26-28] | 25 [24-26] | 0.0001 |
| IQCODE median [p25-p75] | 50 [50-60] | 70 [60-70] | 0.0001 |
| NPI median [p25-p75] | 4 [0-8] | 2 [2-6] | 0.7 |
| BARTHEL INDEX | 100 | 100 | 0.8 |
| IADL median [p25-p75] | 8 [7-8] | 6 [5-7] | 0.001 |
| FA DEMENTIA (%) | 5 (26) | 21 (26) | 0.9 |
| MTA median [p25-p75] | 2 [0-2] | 2 [0-2] | 0.7 |
| FAZEKAS SCHELLE 0 (%)  1 (%)  2 (%) | 9 (47)  8 (42)  2 (10) | 30 (38)  36 (45)  14 (16) | 0.7 |
| PITTSBURG SLEEP Q.I. median [p25-p75] | 6 [2-10] | 4 [2-6] | 0.02 |
| LIVING  In couple (%)  Others (%) | 14 (73)  5 (26) | 48 (61)  32 (39) | 0.3 |
| AMNESTIC MCI (%) | ………. | 78 (98) | …… |
| UPDRS III | 0 | 0 | 0.5 |
| APOE GENOTYPE  ε 3 (%)  ε 4 (%) | 14 (73)  2 (10) | 20 (25)  25 (31) | 0.01 |
| Aβ_42_ pg/mL  median (p25-p75) | 1228  (940-1403) | 627  (508-701) | 0.0001 |
| T-TAU pg/mL  median (p25-p75) | 212  (166-246) | 534  (424-767) | 0.0001 |
| P-TAU_181_ pg/mL  median (p25-p75) | 37  (34-49) | 91  (71-118) | 0.0001 |
| RATIO TTAU/ Aβ_42_  median (p25-p75) | 0.17  (0.12-0.22) | 0.88  (0.68-1.35) | 0.0001 |
| RATIO P-TAU/ Aβ_42_  median (p25-p75) | 0.03  (0.03-0.04) | 0.14  (0.11-0.2) | 0.0001 |
| ALPHA-SYNUCLEIN pg/mL  median (p25-p75) | 950  (515-1582) | 1123  (909-1952) | 0.04 |
| RATIO AS/P-TAU_181_  median (p25-p75) | 23  (14.5-31) | 12.9  (8.6-21) | 0.001 |

Legend for all Supplementary data tables:

AD= Alzheimer´s disease. AS= alpha-sinuclein. PS= psychotic symptoms. FA dementia= familial antecedents of dementia. IADL= instrumental activity daily living. MMSE: minimental state examination. IQCODE= Informant Questionnaire on Cognitive Decline in the Elderly. NPI= Neuropsychiatric examination inventory. PSQI= Pittsburg sleep quality index. UPDRS III= Unified Parkinson disease rating scale. Aβ_42_,= Aβ_42_ protein. T-tau= total tau protein. P-tau_181_= phosphorylated tau 181_181_.
